# Effects of X-Ray Radiation on Complex Visual Discrimination Learning and Social Recognition Memory in Rats

**DOI:** 10.1371/journal.pone.0104393

**Published:** 2014-08-06

**Authors:** Catherine M. Davis, Peter G. Roma, Elwood Armour, Virginia L. Gooden, Joseph V. Brady, Michael R. Weed, Robert D. Hienz

**Affiliations:** 1 Division of Behavioral Biology, Department of Psychiatry and Behavioral Sciences, The Johns Hopkins University School of Medicine, Baltimore, Maryland, United States of America; 2 Department of Radiation Oncology and Molecular Radiation Sciences, The Johns Hopkins University School of Medicine, Baltimore, Maryland, United States of America; 3 Institutes for Behavior Resources, Baltimore, Maryland, United States of America; Sapienza University of Rome, Italy

## Abstract

The present report describes an animal model for examining the effects of radiation on a range of neurocognitive functions in rodents that are similar to a number of basic human cognitive functions. Fourteen male Long-Evans rats were trained to perform an automated intra-dimensional set shifting task that consisted of their learning a basic discrimination between two stimulus shapes followed by more complex discrimination stages (e.g., a discrimination reversal, a compound discrimination, a compound reversal, a new shape discrimination, and an intra-dimensional stimulus discrimination reversal). One group of rats was exposed to head-only X-ray radiation (2.3 Gy at a dose rate of 1.9 Gy/min), while a second group received a sham-radiation exposure using the same anesthesia protocol. The irradiated group responded less, had elevated numbers of omitted trials, increased errors, and greater response latencies compared to the sham-irradiated control group. Additionally, social odor recognition memory was tested after radiation exposure by assessing the degree to which rats explored wooden beads impregnated with either their own odors or with the odors of novel, unfamiliar rats; however, no significant effects of radiation on social odor recognition memory were observed. These data suggest that rodent tasks assessing higher-level human cognitive domains are useful in examining the effects of radiation on the CNS, and may be applicable in approximating CNS risks from radiation exposure in clinical populations receiving whole brain irradiation.

## Introduction

Radiation therapy is a common treatment for numerous cancers in children and adults but it also has deleterious effects on healthy tissue. Radiation-induced cognitive deficits are reported in 50–90% of adult patients receiving therapeutic whole brain irradiations surviving for 6 months or more post-exposure [1]. With the improvement in cancer treatment techniques, this patient population is growing rapidly and there are no effective prevention strategies or successful long-term treatment strategies to mitigate these cognitive deficits.

Numerous preclinical investigations have supported the sensitivity of the hippocampus to radiation-induced damage, given the fact that radiation decreases hippocampal neurogenesis and induces hippocampal-dependent cognitive deficits in rodents [2]. While patients report cognitive deficits associated with both hippocampus- and non-hippocampus-dependent cognitive domains, there is a lack of preclinical data examining the effects of radiation on behaviors mediated by brain regions other than the hippocampus. Thus, there is a need for research focused on tasks related to the function of other brain structures in rodents, in order to further understand radiation’s effects on these other complex cognitive domains reported to be negatively effected by therapeutic radiation in humans.

To date there have been a number of studies of the general behavioral and motor effects of radiation exposure in rodents. Early work examined the effects of gamma sources on motor function and operant performances in rodents [3], [4], [5], [6], [7]. Additionally, high-energy radiation was shown to be more effective in disrupting motor function [8] compared to these conventional sources. Further, Rabin and colleagues have examined the effects of HZE radiation on a number of behavioral processes including conditioned taste aversion, conditioned place preference, emesis, maze performances, and operant responding on a progressive-ratio schedule [9], [10], [11], [12], [13], [14], [15], [16], [17], [18], [19], [20], [21]. Such studies have provided a knowledge base for progressing into the more explicit study of those human cognitive functions likely to be affected by radiation.

A prior report from this laboratory presented data on the effects of gamma irradiation on neurobehavioral function in rodents (psychomotor speed, discrimination accuracy, and inhibitory control) as measured by a simple reaction time (SRT) task [22]. An experimental group was exposed to a single exposure of head-only gamma radiation (5 Gy at a dose rate of 1 Gy/min), while a control group received a sham-radiation exposure using the same anesthesia protocol. Only the irradiated group showed significant deficits in both performance accuracy (as indicated by lower percent correct scores) and performance reliability (as indicated by higher “guessing”, or false alarm rates) from one to four months following radiation, indicating clear performance impairments. The increase in false alarm scores was consistent with reduced inhibitory control, or a shift towards increased anticipatory responses at the cost of decreased accuracy, a common behavioral characteristic of attentional disorders.

The present report extends this research on neurobehavioral function in rats, and centers on demonstrating the effectiveness of the overall methodology and the employment of two additional procedures – an automated intra-dimensional (ID) set shifting task using objective measures of visual stimulus discrimination and reversal via computer-controlled touchscreens – and a social recognition memory task. The ID task is a computerized analog of the Wisconsin card sort task used to test category abstraction, and is similar in function to non-automated tests of set shifting in rats that use odor, texture, or color as stimulus dimensions. Set shifting tasks measure discrimination learning, reversal learning, perseverative responding, and the ability to switch attentional sets between categories (i.e., cognitive flexibility). Traditional set shifting ‘dig tasks’, in which rats must dig through various mediums scented with specific odorants, have demonstrated deficits in complex reversal discrimination performance and intra- and extra-dimensional discrimination learning and the associated reversal following HZE exposure [23].

Thus, testing X-irradiated rats in the current automated ID task provides a more objective assessment of the effects of radiation on attentional set shifting, a behavior mediated by several brain regions, including the medial prefrontal cortex, basal forebrain, and cingulate cortex. Additionally, the effects of X-irradiation were assessed on putative perirhinal cortex-dependent memory [24], [25], and not the more commonly studied hippocampal-dependent memory, by the use of a social recognition memory task that examined the degree to which rats explored wooden beads impregnated with either their own odors or with the odors of novel, unfamiliar rats following repeated exposures to the beads.

## Materials and Methods

### Subjects

Fourteen experimentally naive, Long-Evans hooded male rats (Harlan Sprague-Dawley), received at 6 weeks of age, served as subjects. They were housed individually under a 12∶12 hr light/dark cycle with continuous access to water and with food freely available. Feeding was restricted during shaping of the touchscreen response, but weight gain still was permitted. By the beginning of the ID procedure itself, weights approximated the 340 to 350 g, a range at which body weights for adult male rats of this strain are typically maintained during behavioral studies [26]. Under the ID procedure, rats earned food (45-mg Noyes Precision rat pellets) during the experimental sessions, and were given commercial laboratory rat chow supplements to maintain their weight. When sessions were not conducted, the rats were fed 12–15 g of the rat chow, which typically resulted in weight stability or weight gain on the day the rats were next weighed. Laboratory animal care was according to Public Health Service (PHS) Policy on the Humane Care and Use of Laboratory Animals, and the protocol was approved by the Institutional Animal Care and Use Committee of the Johns Hopkins University. Johns Hopkins also maintains accreditation of their program by the Association for the Assessment and Accreditation of Laboratory Animal Care (AAALAC).

### Behavioral Apparatus

Daily sessions were conducted in standard rat behavioral test cages from Coulbourne Instruments (Whitehall, PA). Each chamber was equipped with one touch screen encompassing the entire front panel, and a food cup for delivery of food pellets located in the center of the opposite wall. Each chamber was further contained in a sound-attenuating isolation chamber equipped with an exhaust fan that provided ventilation as well as background masking noise. Experimental contingencies were controlled by Whiskercontrol and MonkeyCantab behavioral control programs that ran on a Pentium III PC computer.

### Behavioral Procedures

#### Computer Touchscreen Training

All rats were first shaped by the method of successive approximations [27] to touch a large white square on the touch screen to receive two food pellets for each touch. Once rats were reliably responding (i.e., touching inside the white square boundary), the square was progressively reduced in size until rats were responding to a square of the same size employed for subsequent discrimination testing (360×360 pixels). Rats were additionally shaped to press the square when it appeared randomly in different locations on the screen. Once a rat achieved a 70% or greater accuracy level in this latter stage, rats were then trained to discriminate among single components of the ID task stimuli prior to irradiation; however, actual ID testing with the specific combinations of stimuli discussed below was performed post-irradiation only.

#### Intra-Dimensional Set-Shifting Task (ID Task)

This task consisted of a series of six discrimination-learning stages wherein only one of two stimuli presented on the screen was associated with reinforcement [28], [29]. Within any given stage of the task, a pair of stimuli was presented and the same stimulus was associated with reinforcement until the performance criteria were met, i.e., 6 of 7 consecutive trials correct. A 30 s limited hold (LH) for responding to the screen was employed and correct choices were reinforced with two food pellets. Following a correct choice there was a 5 s inter-trial interval (ITI); following an incorrect choice there was a 9 s ITI. A session consisted of a maximum of 60 trials. If a given stage was completed within a session, the program automatically moved to the next stage within the session (e.g., following 6 of 7 consecutive trials correct on the simple discrimination stage (SD), the program moved to the SD reversal stage, or SDRev). If a given stage was not completed by the end of a daily test session, the next session began at that stage with the performance criteria reset. Left and right positions of the stimuli were varied trial by trial in a pseudorandom fashion. To allow repeated testing with novel stimuli for each administration of the test, eight distinct stimulus sets were used. For each presentation, shape stimuli were initially associated with reinforcement.

**Figure 1:**
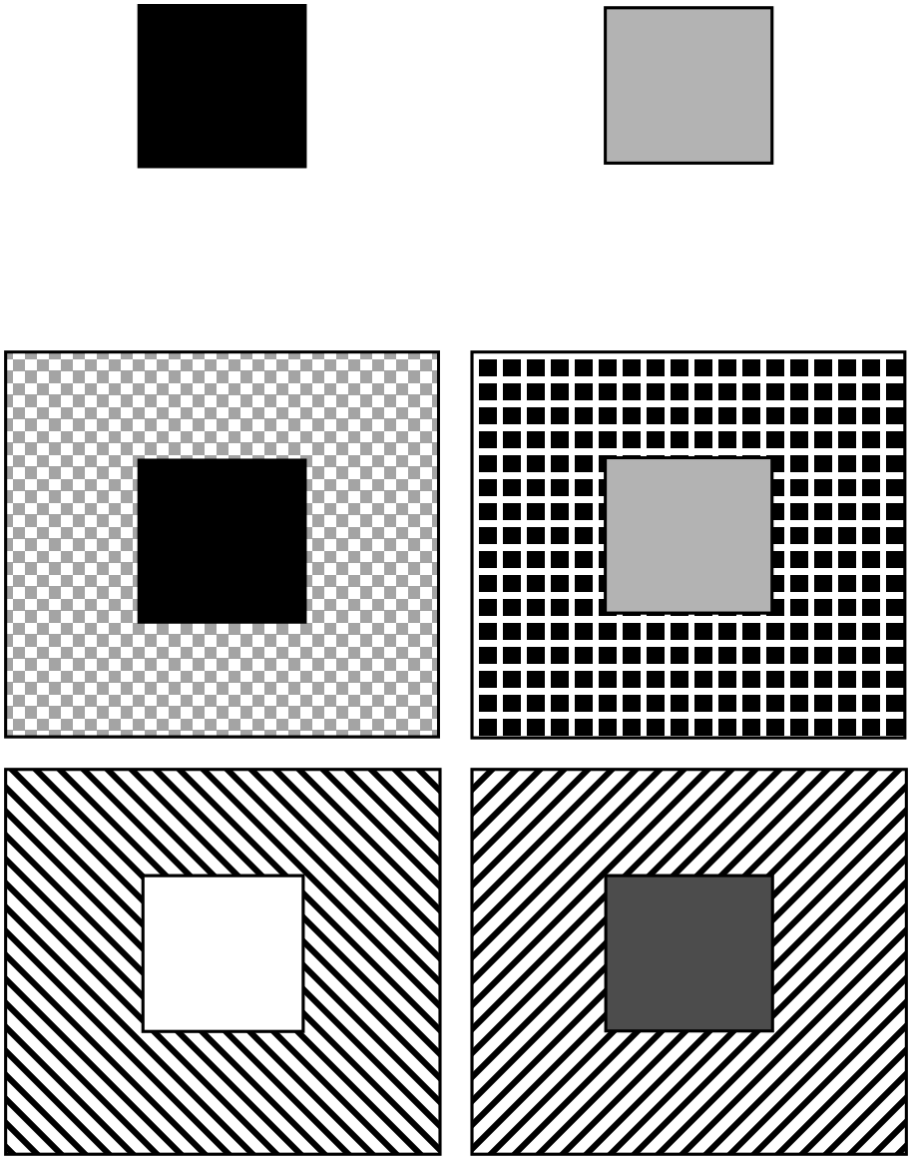
Representative stimuli from the ID touchscreen task. Top row: stimuli used in SD and SDRev. In the SD stage, one square (small dark gray or light gray square) was associated with reinforcement, but during SDRev, the contingencies were reversed and the other square was associated with reinforcement. Middle row: stimuli used during CD and CDRev. In the CD stage, background frames were added to the stimuli. For example, the dark gray square could appear on the gray- or black-checkered frame and could be associated with reinforcement during the CD stage, while during the CDRev stage the light-gray square would appear on the gray- or black-checkered frame and would be associated with reinforcement. Bottom row: stimuli used in IDS and IDSRev. These stages contained new, different shaded squares (i.e., white and a darker gray shade) superimposed on different background frames that continued to be irrelevant. Thus, in this stage rats had to shift within the stimulus dimension of shade of the center square. All stimuli were counterbalanced across rats in both the control and irradiated groups.

Examples of the stimuli used at each stage are diagrammed in Figure 1. The first stage was a simple discrimination (SD) during which responding to one of the two shaded square stimuli was reinforced until the performance criteria were met, at which point the task progressed to the next stage. The second stage was a simple reversal (SDRev) during which the same two stimuli were retained but the reinforcement contingencies were reversed so that responses to the first stimulus were no longer reinforced and responses to the second stimulus were. The third stage was a compound discrimination (CD) that employed the same stimuli and reinforcement conditions as the SDRev stage, but with the addition of line stimuli (either horizontal or vertical) superimposed upon the existing shape stimuli (i.e., compound stimuli of shapes and line orientations). The shape and line stimuli varied left and right positions pseudo-randomly and independently of each other. The fourth stage was a CD reversal (CDRev) in which the reinforcement conditions were reversed (i.e., compound reversal). The fifth stage was an intra-dimensional shift (IDS) discrimination in which a new pair of shape stimuli and a new pair of line stimuli was presented, with one of the shape stimuli associated with reinforcement. This stage was labeled the intra-dimensional shift (IDS) stage because, despite new examples of shape and line stimuli, the same dimension of ‘stimulus shape’ remained relevant for reinforcement. The sixth stage was an IDS reversal discrimination (IDSRev) in which responding to the other shape stimulus was reinforced; line stimuli remained irrelevant to reinforcement in this stage as well.

#### Recognition Memory Task

During an initial 24-h familiarization period, four 2-cm round wooden beads, each with a small hole bored through its diameter were introduced into the home cages in order to acquire the odor of the individual animals and to serve as familiar odors for subsequent use. Several beads were also introduced into the cages of previously selected “odor-donor” animals to provide salient novel odors for the procedure. The selection of “odor-donor” animals’ cages designated to provide donor odor beads were counter-balanced, so that any one odor served as either a recently novel odor (N1) or a brand new novel odor (N2) during memory assessment for different experimental rats.

During the habituation phase of the task, after 24 h of familiarization to the presence of four beads in an animal’s home environment, the four now-familiar beads were removed for 1 h, after which a novel-odor wood bead (N1), taken from an odor-donor cage, and three familiar beads that had been previously taken from an animal’s home cage 1 h previously, were introduced into the cage. Rats were exposed to these four beads for three 1-min trials with 1-min intertrial intervals during which the beads were removed from the testing enclosure. For each 1-min trial, the three familiar-odor beads and the N1 bead were placed in the middle of the testing cage, and the rats were allowed 1 min to actively explore the beads. The first approach to a bead made during this period initiated the timing of the 1-min trial. Exploration time for each of the four beads was recorded and scored by experimenters blinded to which beads were familiar or novel (beads were number-coded). The spatial arrangement of the beads in the middle of the cage was randomly altered between trials. To maximize the sensitivity of the test, one novel (N1) and three familiar-odor beads were used during habituation trials rather than N1 only. Similarly, during memory retention assessment (described below), four beads were used (N1, N2, and two familiar beads) rather than N1 and N2 only. The four-choice procedure for assessing relative odor preference greatly increases power (sensitivity and reliability) compared to two-choice procedures typically used in recognition memory tests.

#### Odor-recognition memory assessment

Twenty-four hours after the novel-odor habituation phase, the odor-recognition test was conducted. For this phase of the task, rats were presented with the odor N1 bead (which it had thoroughly explored on the previous day) in the presence of one unfamiliar novel-odor bead (N2) taken from a different odor-donor cage and two familiar (own-cage) odor beads, following the same procedure outlined for the habituation phase. To dismiss scent marking as a confound, the N1 bead was discarded after habituation and replaced by another N1 bead taken from the same odor-donor cage for the recognition memory phase.

### Data Analysis

At each stage, the basic data collected for the ID procedure were 1) trials to reach criterion, 2) percent trials responded to, 3) number of errors prior to reaching criterion, 4) median response latency, and 5) omitted trials. The standard measure of central tendency employed for the latency data was the median because latency distributions are often skewed due to the physiological limits on response times [30]. Data were analyzed separately for each performance measure with repeated-measures ANOVAs, where Stage (SD, SDRev, CD, CDRev, IDS, and IDSRev) was the repeated-factor and Radiation (Control vs. X-Ray) was the between-subjects factor. When significant effects were found, independent- or paired-samples t-tests with an FDR correction were used to assess specific group differences. Additionally, a version of a ‘survival’ analysis was employed that is commonly used in tactile/olfactory versions of the rodent set-shifting task (i.e., dig task), wherein if an animal does not reach criterion for passing a stage, it is excluded from further analysis of subsequent stages [23]. In the present instance, this was accomplished by excluding any irradiated animal at a particular stage if it did not complete a particular stage in at least the maximum number of trials needed by control rats at that particular stage. This allowed for the calculation of a ‘percent of irradiated rats remaining’ measure, and thus provides a different picture of the irradiated group’s performance as to whether or not they were completing a specific stage in the maximum number of trials needed for control rats to complete that same stage.

For the recognition memory task the basic datum was the amount of time spent sniffing familiar (F1, F2, F3), novel 1 (N1), and novel 2 (N2) objects; these sniffing times were then converted to separate discrimination indices for each trial on Days 1 and 2 in a manner similar to Feinberg et al. [24]. Given that our odor task had four beads, compared to the two beads used in the Feinberg study, DI’ for Day 2 included the familiar beads in the denominator: (sec_N2_–sec_N1_)/(sec_N2_+sec_N1_+sec_F1_+sec_F2_)*100. Separate repeated-measures ANOVAs, with Trial as the repeated-factor and Radiation as the between-subjects factor, were used to determine if radiation affected recognition of the novel beads across trials on Days 1 or 2. When necessary, independent-samples or paired-samples t-tests with an FDR correction were used to assess differences in DI’ on each trial.

### Irradiation Procedure

Once fully trained, rats were transported to Johns Hopkins Radiation Oncology for x-ray radiation exposures via a 225 kVp beam. For the present study, a single 2.3 Gy exposure was administered at a dose rate of 1.9 Gy/min; this single dose was chosen to approximate the single-dose effects previously reported following 1 Gy protons in work from this laboratory [31], [32], [33]. Irradiation involved head-only exposure so that systemic responses to radiation exposures that might confound neurobehavioral testing could be eliminated. The irradiation output of the beam was calibrated with a NIST traceable ionization chamber and electrometer [34]. Dose rate under the “brain” block was evaluated with EBT2 GAFChromic film (International Specialty Products, Wayne, NJ). Rats were anesthetized via i.p. injections of 80 mg/kg of Ketamine (100 mg/mL) in a 10∶1 mixture of Ketamine to Xylazine, and then placed in a holder. Rats in both the irradiated (n = 6) and control (n = 8) groups were identically anesthetized, while only the irradiated group was actually brought into the beam line. After 1 day of recovery the rats were returned to Behavioral Biology for additional behavioral testing starting 7 days post-exposure and continuing until all performance stages were complete (approximately 21–28 days post-exposure).

## Results

### ID Task

**Figure 2:**
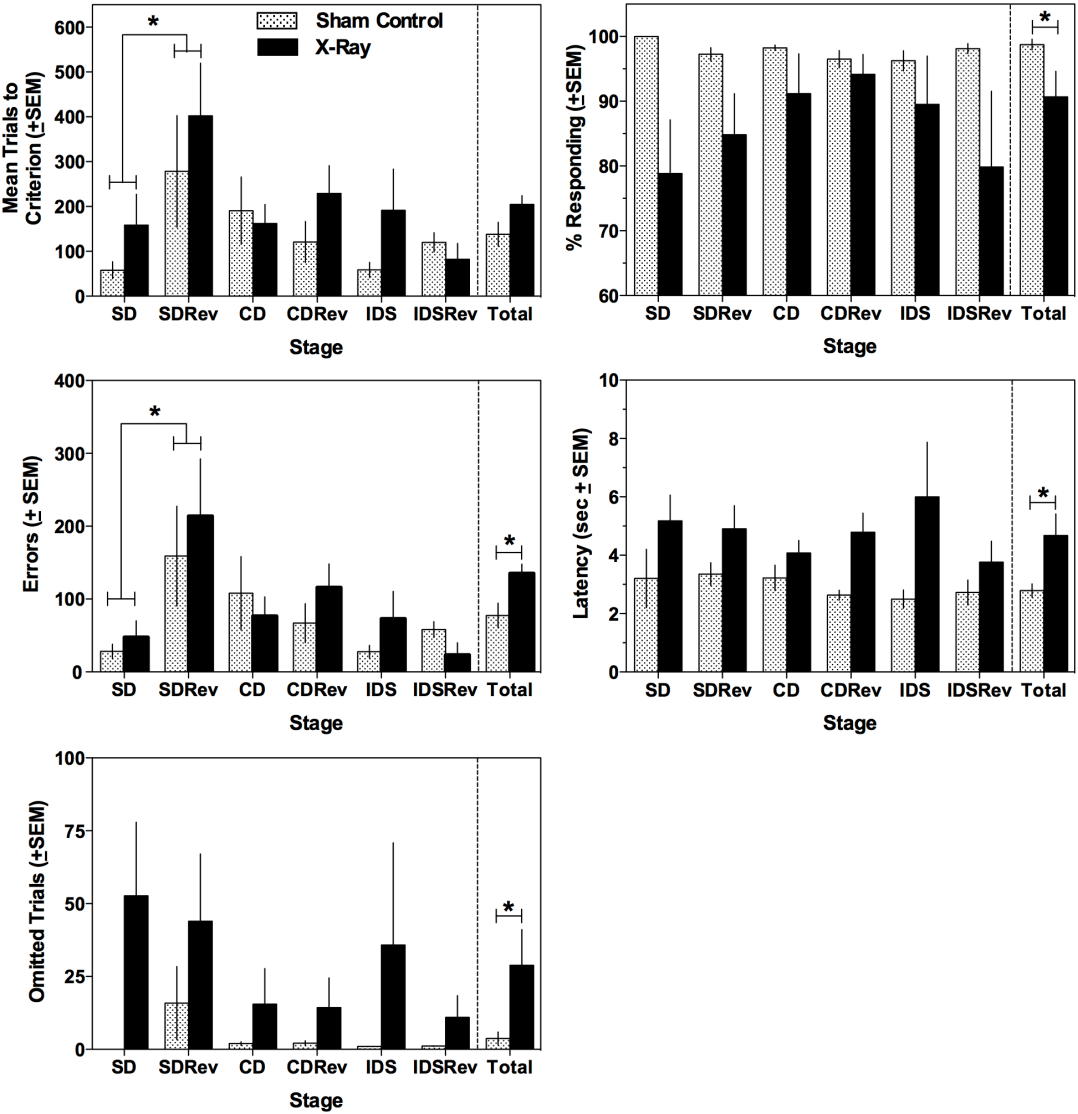
Behavioral changes following x-ray radiation. Mean trials to criterion (top left panel), percentage of trials responded to (top right panel), mean number of errors (middle left panel), median latency to complete each stage (middle right panel), and mean number of omitted trials (bottom left panel) for sham-irradiated control (patterned bars) and all 2.3 Gy X-ray exposed rats (black bars) throughout the stages of the ID task. All rats required more trials to reach criterion and committed more errors on SDRev compared to performances on the SD stage. When collapsed across Stage, irradiated rats responded to significantly fewer trials, committed significantly more errors, displayed significantly longer response latencies, and omitted significantly more trials throughout the entire ID task. The right-most set of bars in each panel, labeled ‘Total’, represent the average of each behavioral parameter collapsed across Stage. *Denotes significant difference, alpha = 0.05.

Figure 2 shows trials to criterion (top left), mean percentage of trials responded to (top right), number of errors (middle left), latency (middle right), and number of omitted trials (bottom left) for the sham-irradiated controls (white bars) and irradiated rats (black bars) at each of the 6 performance stages (SD-IDSRev) as well as group averages collapsed across Stage (last set of bars labeled Total). For mean trials to criterion, a significant within-subjects main-effect of Stage [F(5,60) = 3.116, p = 0.013] was found, but the interaction with Radiation was not significant (p = 0.693), demonstrating that radiation exposure did not increase the number of trials to reach criterion. As expected, significantly more trials were required for all rats to reach criterion on SDRev compared to SD (p = 0.039). Similar numbers of trials were required to meet the criterion between CD and CDRev (p = 0.807) and between IDS and IDSRev (p = 0.841) stages. For percentage of trials responded to, a significant between-subjects effect of Radiation [F(1, 12) = 5.004, p = 0.045] was found, which suggests that collapsed across Stage, irradiated rats responded to fewer trials relative to sham-irradiated controls (see Figure 2). The Stage X Radiation within-subjects interaction approached significance (p = 0.054). In terms of number of errors committed, significant main-effects of Stage [F(5,60) = 5.544, p = 0.0003] and Radiation [F(1,12) = 6.416, p = 0.026] were apparent; however the Stage X Radiation interaction was not significant (p = 0.707). When collapsed across Radiation, all rats committed more errors on SDRev compared to SD (p = 0.018), but errors were similar on CDRev relative to CD (p = 0.461) and on IDSRev relative to IDS (p = 0.537). Irrespective of Stage, irradiated rats, on average, committed more errors than the sham-irradiated control rats. For latency, the between-subjects main-effect of Radiation [F(1,12) = 7.652, p = 0.017] was the only significant effect, demonstrating that when collapsed across Stage, irradiated rats had longer response latencies relative to sham-irradiated controls. Finally, a significant between-subjects main effect of Radiation [F(1,12) = 5.567, p = 0.036] was found for number of omitted trials, such that when collapsed across Stage, irradiated rats omitted more trials relative to sham-irradiated controls.

**Figure 3:**
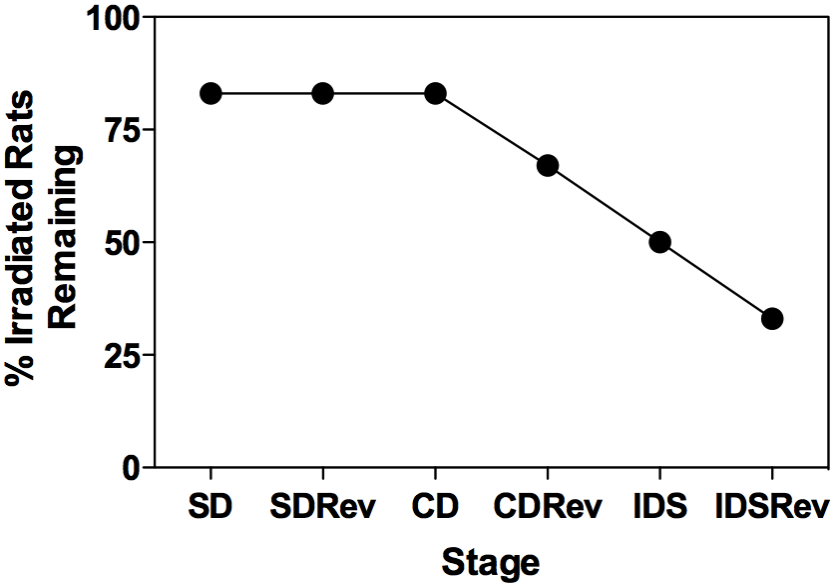
Survival analysis with respect to the effects of exposure on overall discrimination performance. Using the maximum number of trials needed by any control rat as the cutoff point, the percent of irradiated rats remaining at each Stage of the ID task is shown. When evaluated by this method, five of the six irradiated rats reached the standard criterion on Stages 1–3 in the same number of trials or less compared to the control group. Of the remaining rats, four of the six passed CDRev and three of the six passed IDS, whereas only two of the six completed all six Stages of the ID task in the same number of trials or less than all of the control rats.

Figure 3 shows the survival analysis similar to that of Lonart et al [23] with respect to the effects of exposure on overall discrimination performance. At the SD, SDRev, and CD stages, 83.3% (5/6) of the irradiated rats reached the criterion at each stage in less than or equal to the maximum number of trials required by any control rat (160, 993, 678 trials, respectively; see Figure 3). Only 66.6% (4/6) rats successfully reached the CDRev criterion and 50% (3/6) of the irradiated rats reached the IDS criterion by the control maximum for each stage (331 and 151 max control trials, respectively). Two irradiated rats (33.3%) completed the IDSRev by reaching the performance criterion in 207 trials, the maximum number of trials needed by any control rat to complete this stage.

### Recognition Memory Task

Data were first examined to determine whether any differences in baseline rates of exploring the beads were present. While the irradiated group had an initially lower overall rate of bead exploration (“sniffing” time), relative to the control group, the total time spent sniffing the beads was not significantly different between the groups on Day 1 (p = 0.224) or Day 2 (p = 0.437).

**Figure 4:**
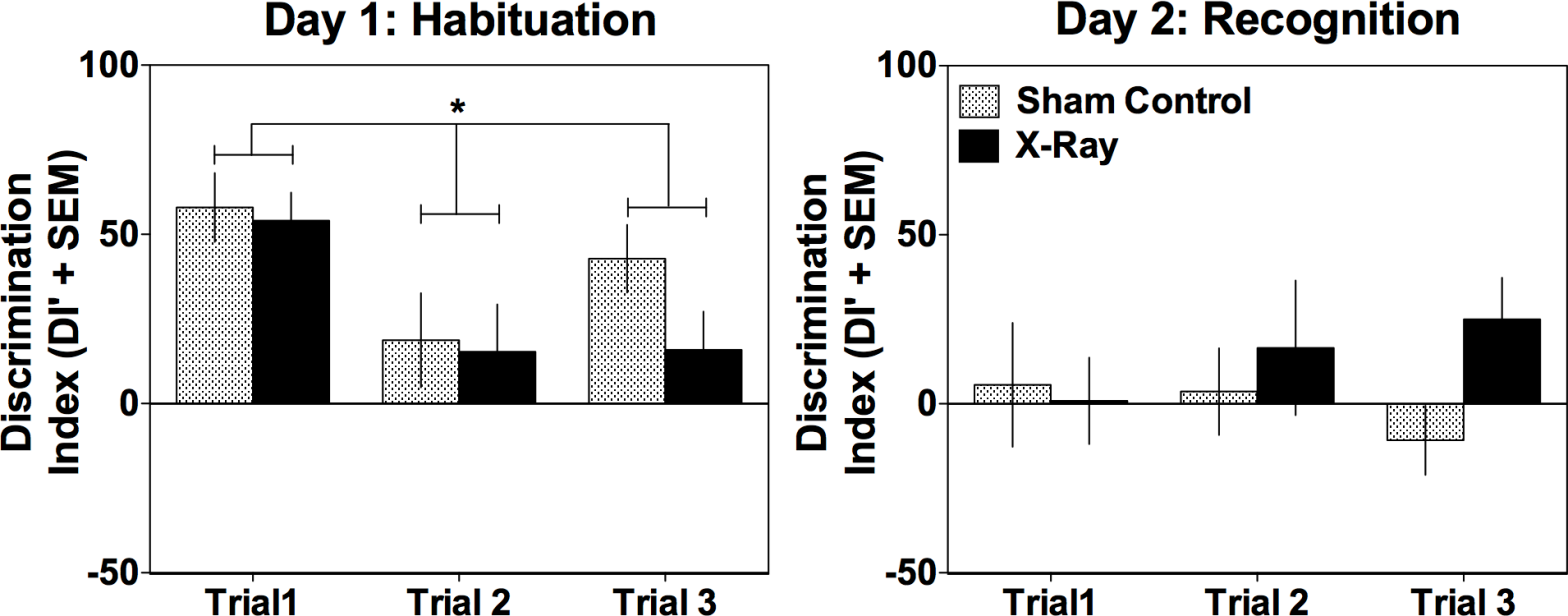
Mean DI’ for sham-control and irradiated rats on the social odor recognition memory task. Data are shown for sham-irradiated control (patterned bar) and 2.3 Gy X-ray exposed rats (black bar) on the habituation day (Day 1, left panel) and on the recognition test day (Day 2, right panel). Irradiated and control rats performed similarly on the Day 1 Habituation trials and displayed a significant preference for the N1 bead on Trial 1, compared to no preference for the N1 bead on Trials 2 and 3. While an increase in DI’ is apparent for the irradiated rats on Trial 3 during the Day 2 Recognition Test, this increase was not significant. *Denotes significant difference, alpha = 0.05.

Figure 4 presents the DI’ across the three trials for both the irradiated and sham control groups on Day 1 (Habituation, left panel) and Day 2 (Recognition, right panel) of the study. The ANOVA on Day 1 revealed a significant within-subjects effect of Trial [F(2, 24) = 7.330, p = 0.003], but no between-subjects effect of Radiation (p = 0.338) or interaction of Trial X Radiation (p = 0.446). All rats displayed a preference for the N1 bead on Trial 1, compared to Trials 2 and 3 (all p’s≤0.007). No difference in DI’ was found between Trials 2 and 3 (p = 0.188), suggesting that all rats habituated to the N1 bead over the Trials 2 and 3.

The ANOVA for Day 2 did not reveal any significant within-subjects effects (Trial: p = 0.922; Trial X Radiation p = 0.498); the between-subjects effect of Radiation showed a trend, but was not significant (p = 0.09).

## Discussion

The present results show that 2.3 Gy of head-only X-irradiation produces deficits in performance on complex visual discriminations, but no significant deficits on social recognition memory. Under the ID visual discrimination procedure, on average the irradiated group responded on fewer trials, committed more errors, displayed longer response latencies, and omitted more trials, compared to the sham controls. However, there were no effects of radiation on the number of trials needed to reach criterion in the visual discrimination task, an effect likely due to the between-subject variability in performance as well as the small sample size. These decreases in overall responding, irrespective of ID task Stage, parallel the decreases in operant response rates seen with whole-body gamma irradiation in various operant performances (e.g., fixed-ratio, fixed-interval, multiple schedules, repeated acquisition) [3], [4], [5], [6], [7]. When the ID task data were analyzed in terms of the ‘percent irradiated rats remaining’, only 2 of the irradiated rats were able to complete all stages of this task in the maximum number of trials needed by any control rat. The social recognition memory tests revealed only slight differences (not significant) in recognition memory performances on Day 2, with the irradiated rats displaying increasing DI’ across trials, which suggests that these rats were displaying an increasing preference for the N2 bead, whereas the sham controls displayed little preference for the N2 bead across trials. When the DI’ for Trial 3 was analyzed with a one-way ANOVA, irradiated rats had a significantly greater DI’ compared to controls (p = 0.044). This difference did not reach statistical significance when analyzed with the repeated-measures ANOVA, which may be due to the small sample size. Irradiation did not impact this group’s ability to learn to distinguish a novel odor from a familiar odor, as was evident by the lack of an effect of x-irradiation on Day 1 DI’.

While acute 2 Gy X-ray exposures, like those in the present study, have been shown to significantly decrease the numbers of proliferating cells and immature neurons in the brains of rodents [35], there are also suggestions in the literature that continuous exposure to learning tasks such as the ID procedure may act to provide an ‘enriched environment’ that stimulates neurogenesis following irradiation, thus possibly reducing potential cognitive deficits [36]. Fan and colleagues (2007) demonstrated the positive effect of environmental enrichment on brain irradiation and neurogenesis following an acute exposure to 10 Gy X-rays, a dose much higher than the 2.3 Gy dose used in the current study; ‘enriched’ animals displayed significant increases in neurogenesis following radiation (compared to animals living in a standard environment) and decreased cognitive deficits. Such an interpretation agrees with the subtle effects of X-irradiation effects on the ID task, which was assessed first following irradiation, and also with the lack of significant effects of radiation on the social recognition memory task that was completed following the conclusion of the possibly enriching ID testing.

While the tasks used in the current study are not thought to explicitly require the hippocampus, there is evidence in the literature regarding the interaction of the hippocampus with numerous areas of the brain involved in performance of these tasks, including the medial prefrontal cortex, basal forebrain, both anterior and posterior cingulate cortices, and the perirhinal cortex [23], [24]. For example, afferent and efferent pathways connect the hippocampus to the medial prefrontal cortex, and while damage alone to this area could impact simple discrimination, the fact that these areas are part of an interconnected network suggests that damage to either area could cause deficits in attentional set shifting. Indeed, hippocampal neurogenesis has been shown to play a role in cognitive flexibility in mice [37], the same cognitive domain assessed in the ID task used in the present study. Further, hippocampal neurogenesis is implicated in psychiatric illnesses such as major depressive disorder and anxiety, in addition to resistance to antidepressant drugs. Such effects are actually mediated by brain regions other than the hippocampus (e.g., the amygdala, nucleus accumbens, and prefrontal cortex) but are dependent upon activation of hippocampal neurogenesis and specific downstream proteins that are associated with cognitive performance, inflammatory disorders, Alzheimer’s disease, and depression [38]. Indeed, several neurotrophic factors important for hippocampal neurogenesis are also implicated in cognition, such as vascular endothelial growth factor (VEGF) and brain-derived neurotrophic factor (BDNF). Interestingly, VEGF antagonists are common cancer treatments and are associated with ‘chemobrain’, an anecdotal term for cognitive decline associated with this antagonist during chemotherapy [39]. More work is needed to fully understand the interaction of the hippocampus and other brain regions, such as the prefrontal cortex, in radiation’s effects on the CNS, in addition to the interaction of these effects with those of chemotherapeutic agents.

In summary, low dose x-irradiation in rats produces deficits in specific aspects of an automated ID task that is analogous to some visually-based cognitive tasks in humans (i.e., employs computer touchscreens and elements of the CANTAB neuropsychological test battery). Future work should assess the effects of higher radiation doses and fractionated whole-brain exposures, in addition to ablation of specific brain areas involved in these tasks via focal irradiation, to further elucidate the radiation-induced damage to these areas and their ability to recover and/or respond to pharmaceutical intervention.

## References

[1] Greene-Schloesser D, Robbins ME (2012) Radiation-induced cognitive impairment–from bench to bedside. Neuro Oncol 14 Suppl 4iv37–44. 23095829 10.1093/neuonc/nos196 PMC3480242

[2] Greene-Schloesser D, Moore E, Robbins ME (2013) Molecular pathways: radiation-induced cognitive impairment. Clin Cancer Res 19: 2294–2300. 23388505 10.1158/1078-0432.CCR-11-2903 PMC3642233

[3] Mele PC, Franz CG, Harrison JR (1988) Effects of sublethal doses of ionizing radiation on schedule-controlled performance in rats. Pharmacol Biochem Behav 30: 1007–1014. 3227026 10.1016/0091-3057(88)90133-5

[4] Mele PC, Franz CG, Harrison JR (1990) Effects of ionizing radiation on fixed-ratio escape performance in rats. Neurotoxicol Teratol 12: 367–373. 2392096 10.1016/0892-0362(90)90056-i

[5] Mele PC, McDonough JH (1995) Gamma radiation-induced disruption in schedule-controlled performance in rats. Neurotoxicology 16: 497–510. 8584281

[6] Winsauer PJ, Bixler MA, Mele PC (1995) Differential effects of ionizing radiation on the acquisition and performance of response sequences in rats. Neurotoxicology 16: 257–269. 7566685

[7] Winsauer PJ, Mele PC (1993) Effects of sublethal doses of ionizing radiation on repeated acquisition in rats. Pharmacol Biochem Behav 44: 809–814. 8469693 10.1016/0091-3057(93)90010-q

[8] Bogo V, Zeman GH, Dooley M (1989) Radiation quality and rat motor performance. Radiat Res 118: 341–352. 2727262

[9] Rabin BM, Buhler LL, Joseph JA, Shukitt-Hale B, Jenkins DG (2002) Effects of exposure to 56Fe particles or protons on fixed-ratio operant responding in rats. J Radiat Res (Tokyo) 43 Suppl: S225–228 10.1269/jrr.43.s225 12793763

[10] Rabin BM, Hunt WA (1986) Mechanisms of radiation-induced conditioned taste aversion learning. Neurosci Biobehav Rev 10: 55–65. 2871536 10.1016/0149-7634(86)90033-3

[11] Rabin BM, Hunt WA (1992) Relationship between vomiting and taste aversion learning in the ferret: studies with ionizing radiation, lithium chloride, and amphetamine. Behav Neural Biol 58: 83–93. 1333765 10.1016/0163-1047(92)90291-b

[12] Rabin BM, Hunt WA, Joseph JA (1989) An assessment of the behavioral toxicity of high-energy iron particles compared to other qualities of radiation. Radiat Res 119: 113–122. 2756102

[13] Rabin BM, Hunt WA, Lee J (1988) Attenuation and cross-attenuation in taste aversion learning in the rat: studies with ionizing radiation, lithium chloride and ethanol. Pharmacol Biochem Behav 31: 909–918. 2855272 10.1016/0091-3057(88)90404-2

[14] Rabin BM, Hunt WA, Wilson ME, Joseph JA (1992) Emesis in ferrets following exposure to different types of radiation: a dose-response study. Aviat Space Environ Med 63: 702–705. 1510644

[15] Rabin BM, Joseph JA, Hunt WA, Dalton TB, Kandasamy SB, et al (1994) Behavioral endpoints for radiation injury. Adv Space Res 14: 457–466. 11539983 10.1016/0273-1177(94)90500-2

[16] Rabin BM, Joseph JA, Shukitt-Hale B (2004) Heavy particle irradiation, neurochemistry and behavior: thresholds, dose-response curves and recovery of function. Adv Space Res 33: 1330–1333. 15803623 10.1016/j.asr.2003.09.051

[17] Rabin BM, Joseph JA, Shukitt-Hale B (2005) A longitudinal study of operant responding in rats irradiated when 2 months old. Radiat Res 164: 552–555. 16187786 10.1667/rr3349.1

[18] Rabin BM, Joseph JA, Shukitt-Hale B (2005) Effects of age and diet on the heavy particle-induced disruption of operant responding produced by a ground-based model for exposure to cosmic rays. Brain Res 1036: 122–129. 15725409 10.1016/j.brainres.2004.12.041

[19] Rabin BM, Shukitt-Hale B, Joseph JA, Denissova N (2001) Effects of exposure to 56Fe particles on the acquisition of a conditioned place preference in rats. Phys Med 17 Suppl 1196–197. 11776260

[20] Shukitt-Hale B, Casadesus G, Cantuti-Castelvetri I, Rabin BM, Joseph JA (2003) Cognitive deficits induced by 56Fe radiation exposure. Adv Space Res 31: 119–126. 12577981 10.1016/s0273-1177(02)00878-5

[21] Shukitt-Hale B, Casadesus G, McEwen JJ, Rabin BM, Joseph JA (2000) Spatial learning and memory deficits induced by exposure to iron-56-particle radiation. Radiat Res 154: 28–33. 10856962 10.1667/0033-7587(2000)154[0028:slamdi]2.0.co;2

[22] Hienz RD, Brady JV, Gooden VL, Vazquez ME, Weed MR (2008) Neurobehavioral effects of head-only gamma radiation exposure in rats. Radiation Research 170: 292–298. 18763858 10.1667/RR1222.1

[23] Lonart G, Parris B, Johnson AM, Miles S, Sanford LD, et al (2012) Executive function in rats is impaired by low (20 cGy) doses of 1 GeV/u (56)Fe particles. Radiat Res 178: 289–294. 22880624 10.1667/rr2862.1

[24] Feinberg LM, Allen TA, Ly D, Fortin NJ (2012) Recognition memory for social and non-social odors: differential effects of neurotoxic lesions to the hippocampus and perirhinal cortex. Neurobiol Learn Mem 97: 7–16. 21930227 10.1016/j.nlm.2011.08.008

[25] Brown MW, Aggleton JP (2001) Recognition memory: what are the roles of the perirhinal cortex and hippocampus? Nat Rev Neurosci 2: 51–61. 11253359 10.1038/35049064

[26] Ator NA (1991) Subjects and instrumentation. In: Iversen H, Lattal KA, editors. Techniques in the behavioral and neural sciences: Experimental analysis of behavior. Amsterdam: Elsevier. 1–62.

[27] Skinner BF (1938) The behavior of organisms: an experimental analysis. New York: Appleton-Century-Crofts. 457 p.

[28] Roberts AC (1996) Comparison of cognitive function in human and non-human primates. Cogn Brain Res 3: 319–327. 10.1016/0926-6410(96)00017-1 8806033

[29] Roberts AC, DeSalvia MA, Wilkinson LS, Collins P, Muir JL, et al (1994) 6-Hydroxydopamine lesions of the prefrontal cortex in monkeys enhance performance on an analog of the Wisconsin card sort test: possible interactions with subcortical dopamine. J Neurosci 14: 2531–2544. 8182426 10.1523/JNEUROSCI.14-05-02531.1994 PMC6577476

[30] Stebbins WC, Miller JM (1964) Reaction time as a function of stimulus intensity for the monkey. Journal of the Experimental Analysis of Behavior 7: 309–312. 14176278 10.1901/jeab.1964.7-309 PMC1404245

[31] Davis CM, Decicco-Skinner KL, Roma PG, Hienz RD (2014) Individual differences in attentional deficits and dopaminergic protein levels following exposure to proton radiation. Radiat Res 181: 258–271. 24611657 10.1667/RR13359.1

[32] Davis CM, Roma PG, Guida PM, Hienz RD (2012) Comparing the effects of 56Fe and proton radiation on psychomotor vigilance. NASA Human Research Performance Investigators’ Workshop. Houston, TX.

[33] Davis CM, Roma PG, Guida PM, Hienz RD (2013) Comparing the effects of ^28^Si, ^56^Fe, and proton radiation on psychomotor vigilance. NASA Human Research Performance Investigators’ Workshop, 2013. Houston, TX.

[34] Wong J, Armour E, Kazanzides P, Iordachita I, Tryggestad E, et al (2008) High-resolution, small animal radiation research platform with x-ray tomographic guidance capabilities. Int J Radiat Oncol Biol Phys 71: 1591–1599. 18640502 10.1016/j.ijrobp.2008.04.025 PMC2605655

[35] Mizumatsu S, Monje ML, Morhardt DR, Rola R, Palmer TD, et al (2003) Extreme sensitivity of adult neurogenesis to low doses of X-irradiation. Cancer Res 63: 4021–4027. 12874001

[36] Fan Y, Liu Z, Weinstein PR, Fike JR, Liu J (2007) Environmental enrichment enhances neurogenesis and improves functional outcome after cranial irradiation. Eur J Neurosci 25: 38–46. 17241265 10.1111/j.1460-9568.2006.05269.x

[37] Burghardt NS, Park EH, Hen R, Fenton AA (2012) Adult-born hippocampal neurons promote cognitive flexibility in mice. Hippocampus 22: 1795–1808. 22431384 10.1002/hipo.22013 PMC4784987

[38] Samuels BA, Hen R (2011) Neurogenesis and affective disorders. Eur J Neurosci 33: 1152–1159. 21395859 10.1111/j.1460-9568.2011.07614.x

[39] Ng T, Cheung YT, Ng QS, Ho HK, Chan A (2014) Vascular endothelial growth factor inhibitors and cognitive impairment: evidence and controversies. Expert Opin Drug Saf 13: 83–92. 23931162 10.1517/14740338.2013.828034

